# Extensive Flexural and Pan-Ungual Involvement in Hallopeau-Type Pemphigus Vegetans: A Case Report

**DOI:** 10.7759/cureus.115333

**Published:** 2026-08-27

**Authors:** Meryeme Marhraoui, Meryem El Moustaoui, Mariame Meziane, Nadia Ismaili, Laila Benzekri

**Affiliations:** 1 Dermatology, University Hospital Center Ibn Sina/Mohamed V University of Rabat, Rabat, MAR

**Keywords:** acantholysis, autoimmune bullous disease, pemphigus vegetans, pemphigus vulgaris, vegetating plaques

## Abstract

Pemphigus vegetans is a rare variant of pemphigus vulgaris characterized by vegetating plaques predominantly involving intertriginous areas. A 34-year-old woman presented with persistent painful oral lesions followed by progressive flexural, palmoplantar, and pan-ungual involvement. Examination revealed exuberant erosive vegetating plaques of the major folds, associated with oral erosions, palmoplantar pustules, and diffuse nail involvement. Histopathology showed papillomatosis, acanthosis, suprabasal cleavage, and acantholysis. Direct immunofluorescence demonstrated intercellular immunoglobulin G and complement C3 deposits, while indirect immunofluorescence detected circulating anti-intercellular substance antibodies, confirming Hallopeau-type pemphigus vegetans. Treatment with systemic corticosteroids and rituximab resulted in progressive healing of the cutaneous and mucosal lesions, with subsequent nail regrowth. This case highlights the importance of considering pemphigus vegetans in patients with vegetating lesions of the folds associated with mucosal involvement and extensive pan-ungual involvement, a rare but clinically distinctive feature of this disease.

## Introduction

Pemphigus vegetans is an uncommon clinicopathologic variant of pemphigus vulgaris, accounting for approximately 1%-2% of pemphigus cases [1]. It is characterized by vegetating, papillomatous plaques with a predilection for intertriginous, perioral, and anogenital regions [2].

Two clinical variants have been described. The Neumann type usually begins with flaccid bullae or erosions that subsequently evolve into vegetating plaques, whereas the Hallopeau type typically begins with pustules that coalesce and evolve into vegetating plaques [2,3]. Because its exuberant vegetating morphology may mimic infectious, inflammatory, or neoplastic dermatoses, diagnosis can be challenging and may be delayed [2-4].

Despite its long-recognized clinical description, pemphigus vegetans remains exceptionally uncommon. A recent systematic review identified 197 patients across 148 published studies [5]. Nail involvement has been described in pemphigus vegetans, with manifestations including paronychia, onycholysis, and onychomadesis; however, diffuse pan-ungual involvement affecting all 20 nails appears to be exceptionally rare [6,7].

We report a case of Hallopeau-type pemphigus vegetans characterized by exuberant flexural vegetations, extensive mucosal involvement, and diffuse pan-ungual involvement affecting all 20 nails, highlighting an unusual manifestation within the clinical spectrum of this rare disease.

## Case presentation

A 34-year-old woman with no significant medical history presented with a two-month history of painful oral lesions associated with erosive cheilitis, tongue burning, and conjunctivitis. She was initially treated with an unspecified topical antibiotic, systemic antibiotics, and antifungal agents. The conjunctivitis resolved, but the oral lesions persisted. No cutaneous microbiological cultures performed before this treatment were documented in the available medical records.

One month later, she developed palmoplantar pustules followed by progressive vegetating lesions involving the axillary, inguinal, pubic, and intergluteal regions, together with diffuse nail involvement. She was subsequently referred to our dermatology department.

The patient was afebrile and hemodynamically stable. Dermatologic examination revealed multiple large, confluent, exophytic vegetating plaques arising on an erythematous base, with prominent verrucous, papillomatous, cauliflower-like surfaces and moist erosive borders. The lesions predominantly involved both axillae and the pubic region, with additional involvement of the inguinal folds, buttocks, and intergluteal cleft (Figures 1A, 1B). Palmoplantar pustules on an erythematous base and crusted erosions were also present.

**Figure 1:**
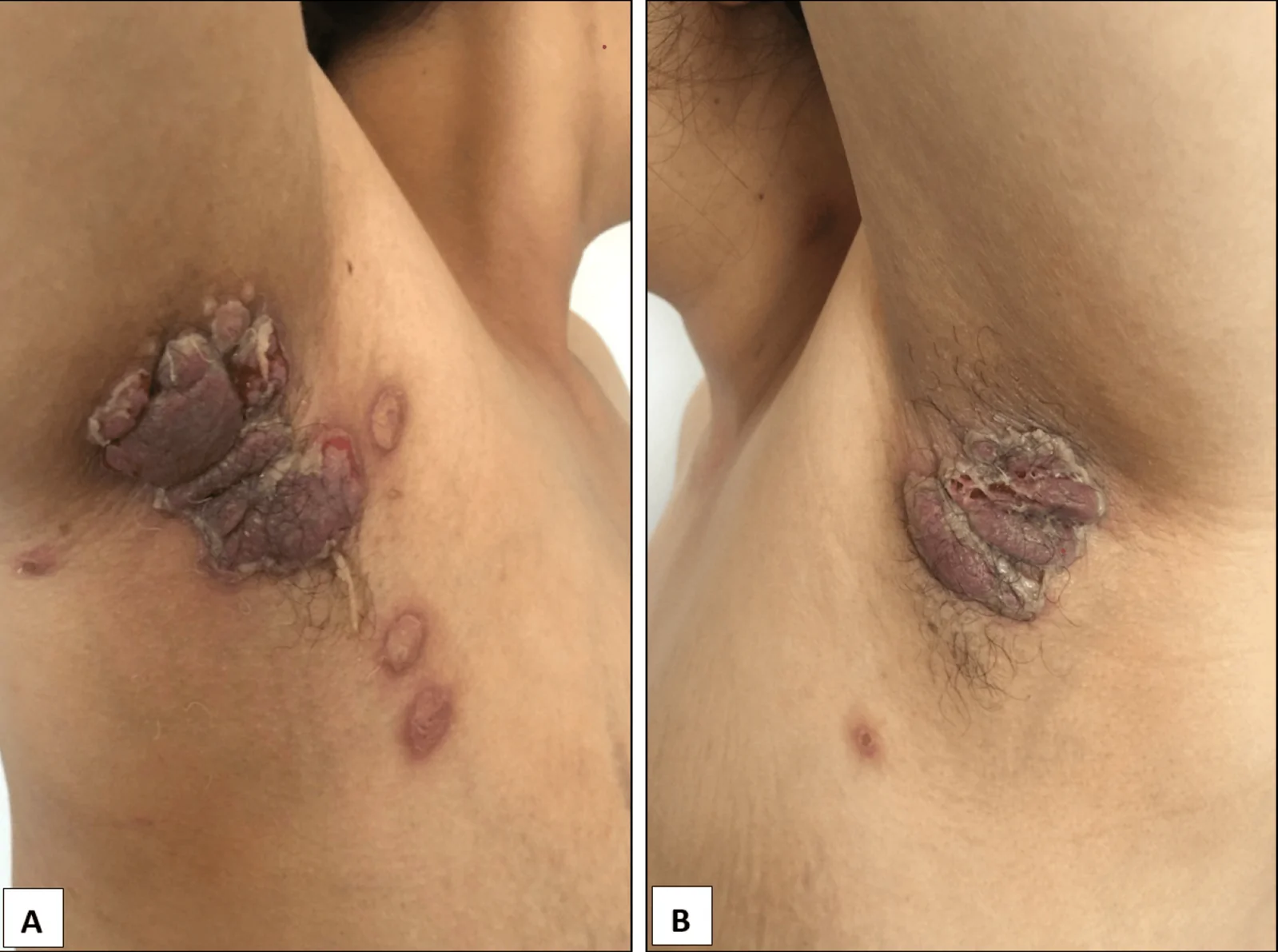
Axillary involvement in Hallopeau-type pemphigus vegetans. (A, B) Exuberant exophytic, papillomatous, and erosive vegetating plaques involving both axillary folds.

All 20 nails showed periungual inflammation and pustules, onycholysis, and onychomadesis, affecting both the fingernails and toenails (Figures 2A, 2B).

**Figure 2:**
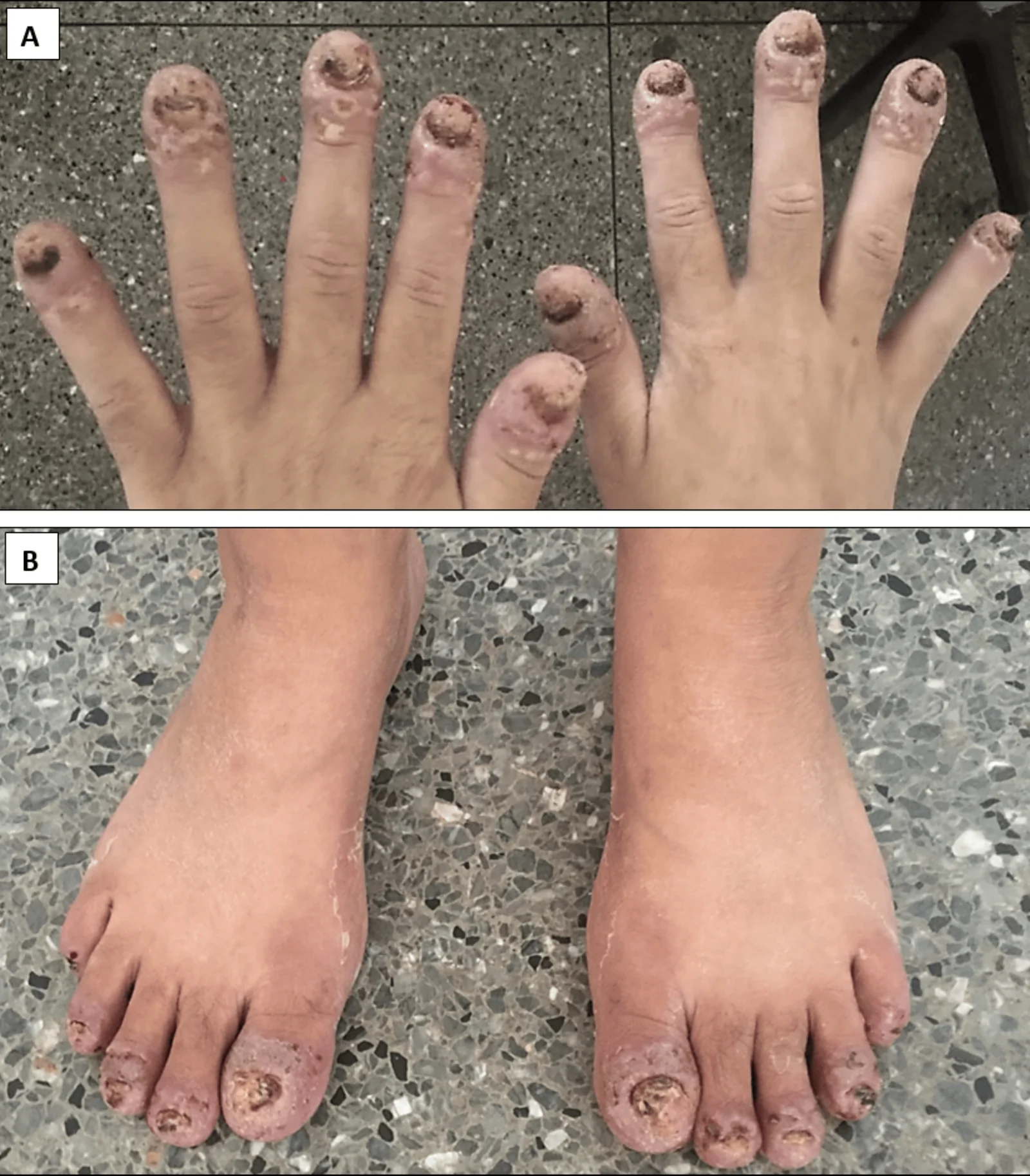
Pan-ungual involvement at presentation. (A) Fingernails showing periungual inflammation and pustules, onycholysis, and onychomadesis. (B) Similar involvement of the toenails.

Mucosal examination revealed erosive cheilitis, extensive oral erosions, and a bright-red depapillated tongue (Figure 3). Genital examination showed mucosal erythema associated with anal fissures.

**Figure 3:**
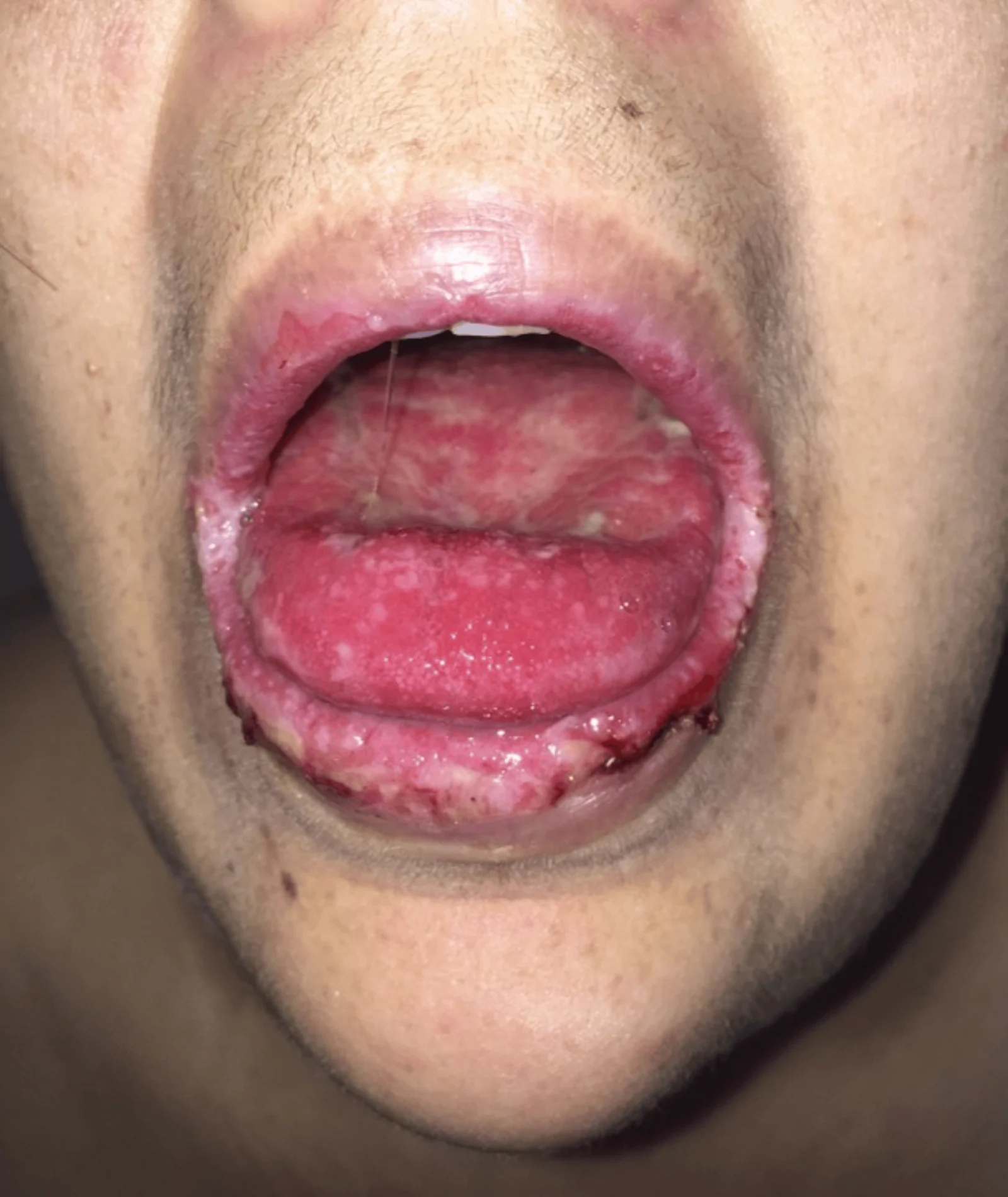
Oral mucosal involvement in Hallopeau-type pemphigus vegetans. Erosive cheilitis, extensive oral erosions, and a bright-red depapillated tongue.

Laboratory investigations revealed eosinophilia, an elevated C-reactive protein level, and hypoalbuminemia. Serologic tests for HIV, hepatitis B and C viruses, and syphilis were negative. The main laboratory and serologic findings are summarized in Table 1.

**Table 1:** Laboratory and Serologic Findings Abbreviations: CRP, C-reactive protein; HIV, human immunodeficiency virus; HBV, hepatitis B virus; HCV, hepatitis C virus; VDRL, Venereal Disease Research Laboratory; TPHA, Treponema pallidum hemagglutination assay.

Laboratory test	Result	Reference range/value	Unit	Interpretation
Hemoglobin	14.1	12.0–16.0	g/dL	Normal
White blood cell count	7.44	4.0–10.0	G/L	Normal
Platelet count	330	150–400	G/L	Normal
Eosinophil count	1.10	0.10–0.50	G/L	Elevated
CRP	46.7	<6	mg/L	Elevated
Serum creatinine	0.68	0.5-1	mg/dL	Normal
Aspartate aminotransferase	20	10–35	IU/L	Normal
Alanine aminotransferase	11	7–35	IU/L	Normal
Total serum protein	58	64–83	g/L	Decreased
Serum albumin	30	35–52	g/L	Decreased
HIV serology	Negative	Negative	—	Negative
HBV serology	Negative	Negative	—	Negative
HCV serology	Negative	Negative	—	Negative
VDRL	Negative	Negative	—	Negative
TPHA	Negative	Negative	—	Negative

Histopathological examination showed verrucous epidermal hyperplasia with acanthosis and papillomatosis, suprabasal clefting with numerous acantholytic cells, and scattered eosinophils and neutrophils within the cleft. A moderate superficial dermal inflammatory infiltrate was also present (Figures 4, 5).

**Figure 4:**
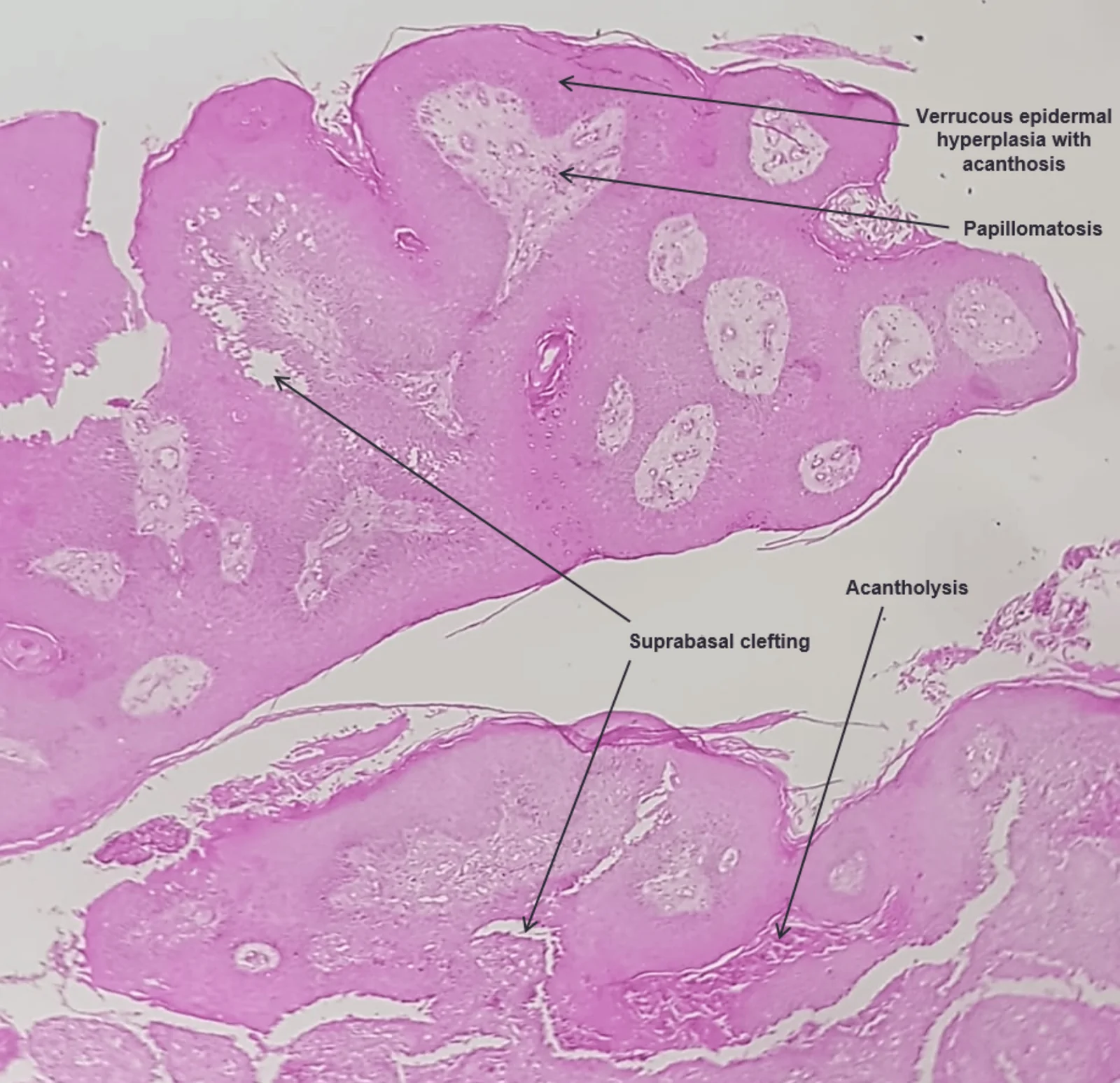
Histopathological findings of pemphigus vegetans. Histopathological examination of a vegetating lesion showing verrucous epidermal hyperplasia with acanthosis, papillomatosis, suprabasal clefting, and acantholysis (hematoxylin and eosin staining, ×40).

**Figure 5:**
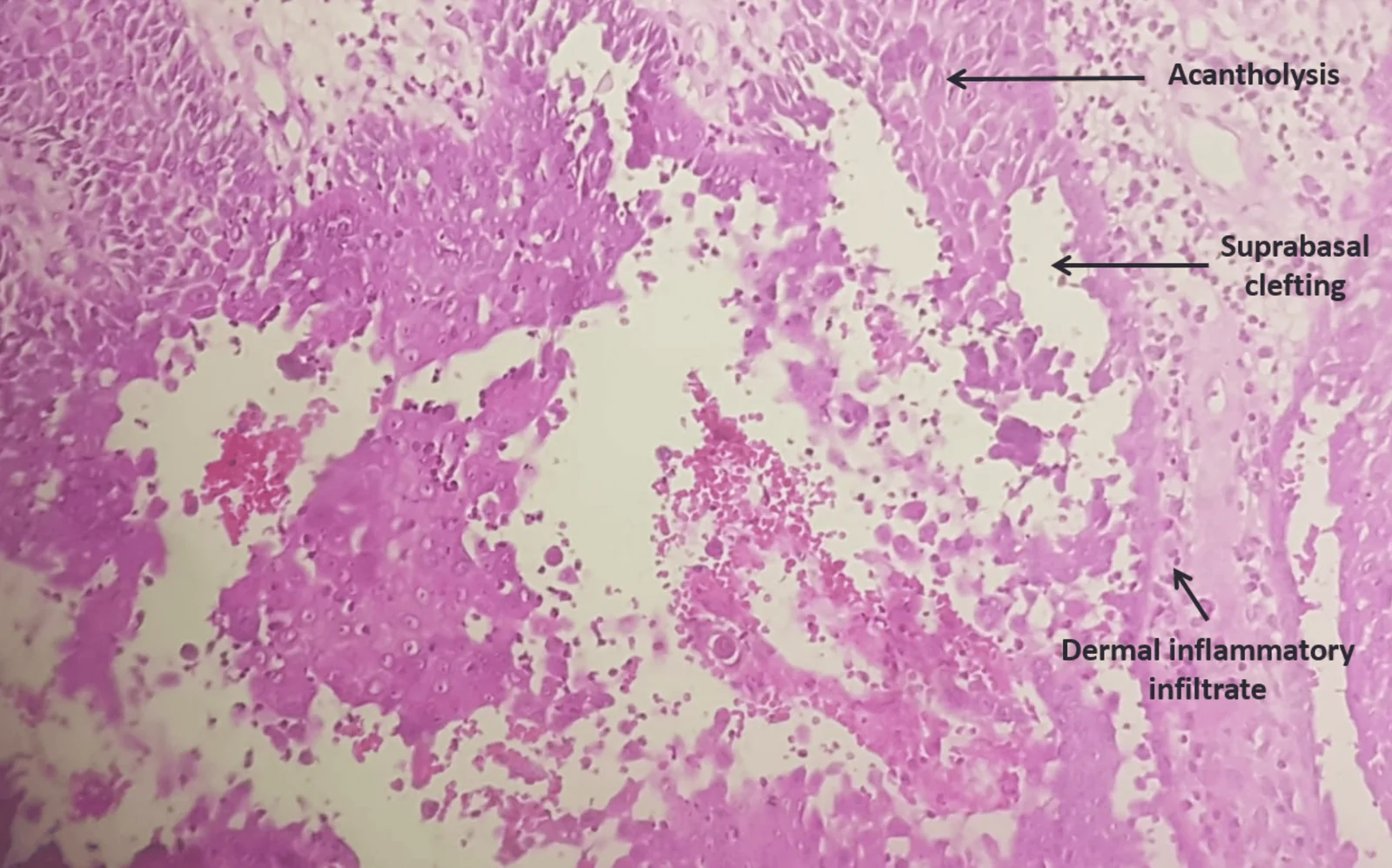
Higher-magnification histopathological findings of pemphigus vegetans. Hematoxylin and eosin staining showing suprabasal clefting, acantholysis, and a dermal inflammatory infiltrate (H&E, ×200).

Direct immunofluorescence revealed intercellular IgG and C3 deposition throughout the epidermis in a characteristic fish-net pattern (Figure 6). Indirect immunofluorescence detected circulating anti-intercellular substance antibodies at a titer of 1:1280. Anti-desmoglein 1 and anti-desmoglein 3 antibody testing was not performed because of financial constraints. These clinical, histopathological, and immunopathological findings established the diagnosis of Hallopeau-type pemphigus vegetans.

**Figure 6:**
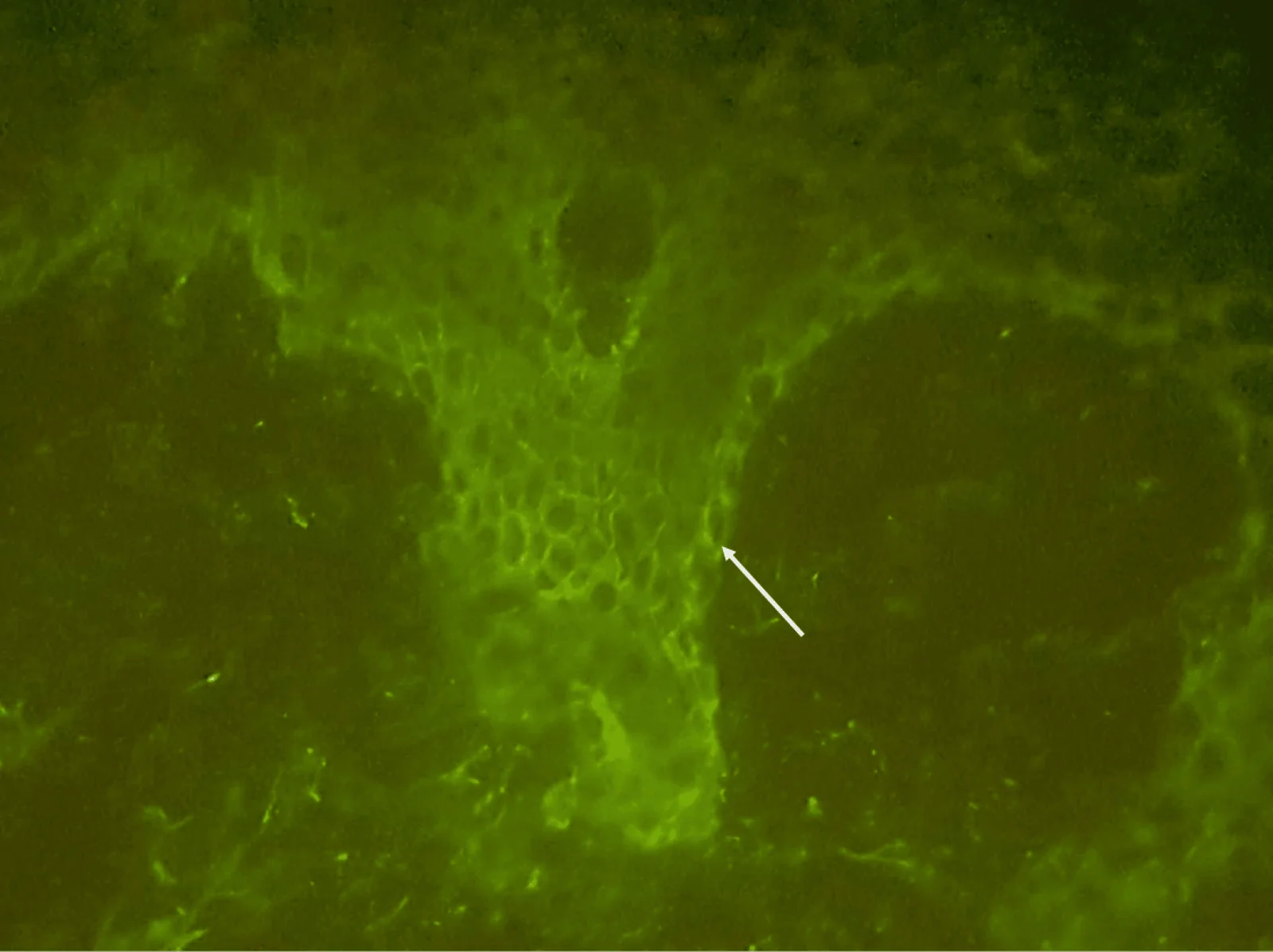
Direct immunofluorescence findings. Intercellular deposition of IgG and C3 throughout the epidermis in a characteristic fish-net pattern (arrow; ×200).

Given the extensive mucocutaneous involvement, treatment with systemic corticosteroids in combination with rituximab was planned. Oral prednisone was initiated at 2 mg/kg/day to achieve rapid disease control while the pre-rituximab evaluation was being completed. Pre-rituximab assessment included a complete blood count, renal and hepatic function tests, C-reactive protein, urinalysis, electrocardiography, chest imaging, viral serologies, tuberculosis screening, and lymphocyte subset analysis. Hepatitis B, hepatitis C, and HIV serologies were negative, tuberculosis screening was negative, and chest imaging was unremarkable. Once the required screening had been completed, rituximab was initiated according to our departmental protocol for pemphigus, with 1-g intravenous infusions on days 1 and 15, followed by 500-mg maintenance infusions at months 6, 12, and 18. Following rituximab initiation, the corticosteroid dose was reduced to 1.5 mg/kg/day and subsequently tapered according to clinical response and the absence of new lesions. Premedication included paracetamol, an antihistamine, and intravenous hydrocortisone, and the rituximab infusions were well tolerated. Adjunctive management included topical wound-healing care with emollient and reparative creams, oral care, nutritional support, and supplementation with calcium, vitamin D, and potassium.

The vegetating and erosive lesions progressively regressed over approximately 10 weeks, leaving residual post-inflammatory hyperpigmentation (Figure 7B). At six months, disease activity remained controlled, with no reappearance of vegetating lesions and further fading of the residual pigmentation (Figure 7C).

**Figure 7:**
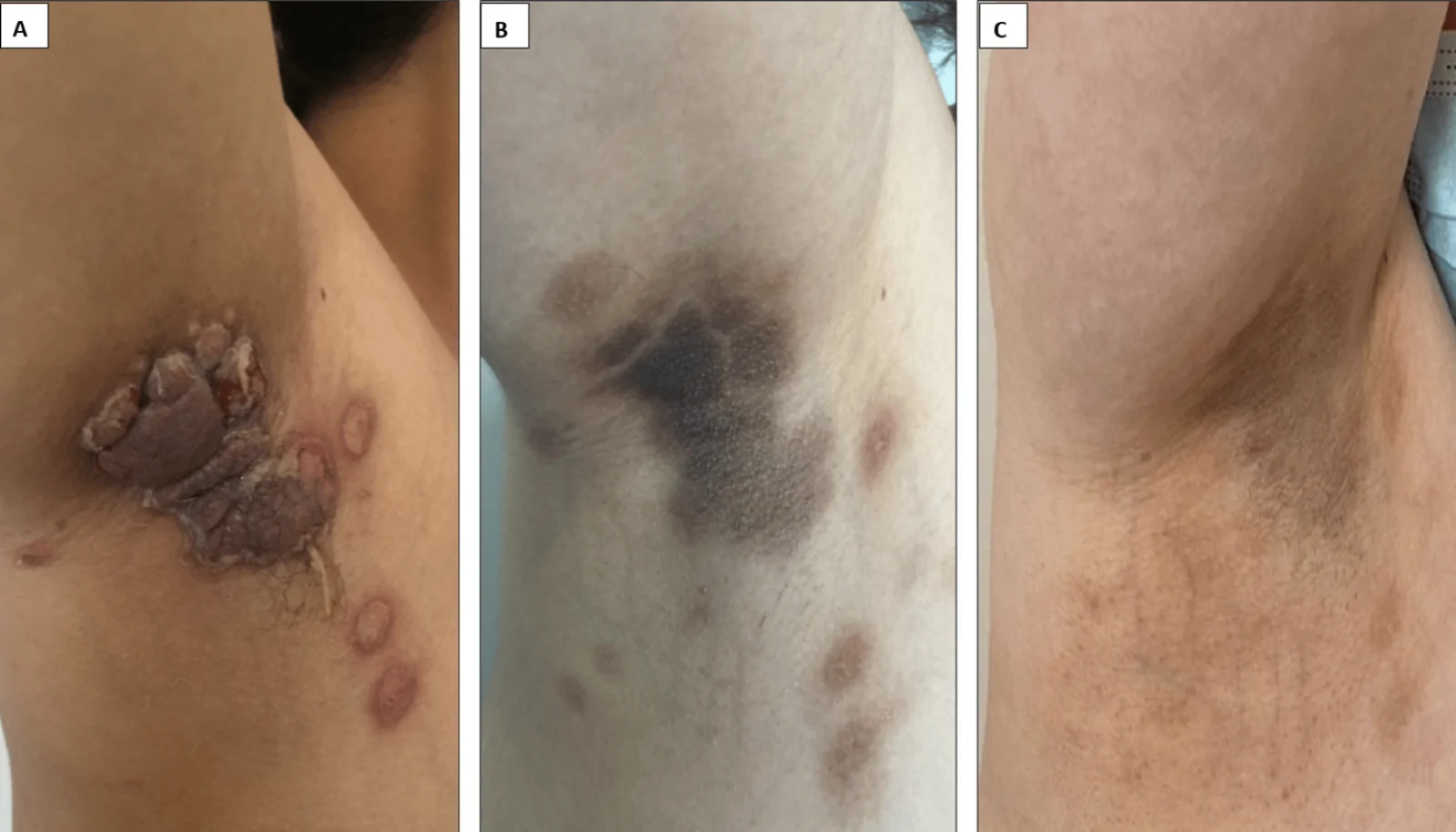
Clinical evolution of the axillary involvement following treatment. (A) Exuberant vegetating axillary plaque at presentation. (B) Marked regression of the vegetating lesion with residual post-inflammatory hyperpigmentation after approximately 10 weeks of treatment. (C) Six-month follow-up showing sustained disease control, with no recurrence of vegetating lesions and marked fading of the residual hyperpigmentation.

The palmoplantar pustules and periungual inflammation resolved, with marked regrowth of the affected fingernails and toenails at six months (Figures 8A, 8B). Systemic corticosteroids were gradually tapered. At the 18-month follow-up, disease remained clinically controlled without relapse, and during follow-up, the indirect immunofluorescence titer decreased from 1:1280 to 1:160.

**Figure 8:**
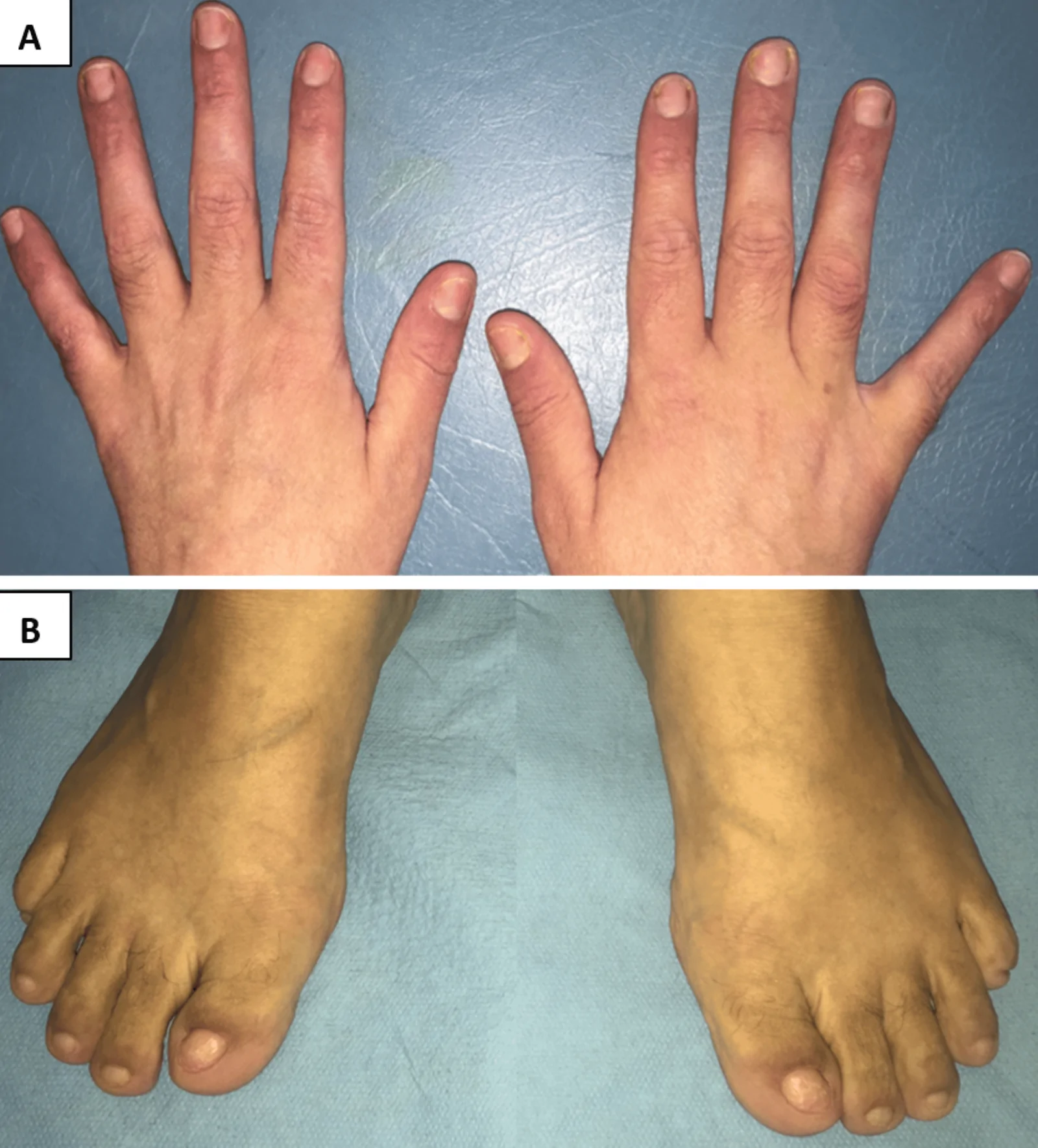
Evolution of nail involvement following treatment. Six-month follow-up showing marked regrowth of the fingernails (A) and toenails (B).

## Discussion

Pemphigus vegetans is a rare form of pemphigus characterized by the development of vegetating plaques, particularly in intertriginous and anogenital areas [1,2]. Similar to pemphigus vulgaris, it is associated with autoantibodies against desmosomal adhesion proteins, principally desmoglein 3 and, in some patients, desmoglein 1. Disruption of keratinocyte adhesion produces suprabasal acantholysis and intraepidermal cleavage [8].

The clinical distinction between the Hallopeau and Neumann variants is based mainly on the morphology of the initial lesions. The Neumann type begins with flaccid bullae or erosions that subsequently evolve into vegetating plaques. In contrast, the Hallopeau type begins with pustules that enlarge or coalesce before becoming vegetating lesions [2,3]. Although oral mucosal erosions preceded the cutaneous manifestations in our patient, this did not preclude classification as the Hallopeau variant. The subsequent pustular-to-vegetating evolution of the cutaneous lesions, without a preceding cutaneous bullous phase, supported the Hallopeau subtype.

The preferential involvement of the axillary, inguinal, pubic, and intergluteal regions was characteristic of pemphigus vegetans [1,2]. The combination of extensive flexural vegetations with mucosal disease and involvement of all 20 nails was particularly striking. Nail abnormalities in pemphigus vegetans may include paronychia, periungual inflammation or pustules, onycholysis, onychomadesis, and nail dystrophy [6,7]. In our patient, diffuse nail involvement with periungual pustules, onycholysis, and onychomadesis represented an additional notable feature of the extensive mucocutaneous disease.

The differential diagnosis included pyodermatitis-pyostomatitis vegetans, secondary syphilis with condylomata lata, Hailey-Hailey disease, and other infectious or inflammatory vegetating disorders [9,10]. The lack of response to antibacterial and antifungal therapy argued against a primary infectious process. Secondary syphilis was also considered because condylomata lata may involve intertriginous and anogenital areas; negative syphilis serology made this diagnosis unlikely [10]. Ultimately, suprabasal acantholysis on histopathology, together with the characteristic intercellular deposition of IgG and C3 on direct immunofluorescence and circulating anti-intercellular substance antibodies on indirect immunofluorescence, confirmed the diagnosis of pemphigus vegetans.

Treatment of pemphigus vegetans generally follows the therapeutic principles established for pemphigus vulgaris. Systemic corticosteroids provide rapid control of disease activity, while rituximab, an anti-CD20 monoclonal antibody targeting B lymphocytes, is a first-line therapeutic option for moderate-to-severe pemphigus [11]. The combination of rituximab with systemic corticosteroids has demonstrated greater efficacy in achieving complete remission while reducing cumulative corticosteroid exposure compared with corticosteroid therapy alone [12]. In our patient, systemic corticosteroids were initially used to rapidly control the extensive mucocutaneous disease, followed by the addition of rituximab and progressive corticosteroid tapering according to clinical response. This therapeutic strategy resulted in sustained control of the cutaneous and mucosal disease, together with progressive improvement of the associated nail abnormalities.

A limitation of this case is that anti-desmoglein 1 and anti-desmoglein 3 antibody testing was not performed because of financial constraints, limiting the immunologic characterization of the disease.

This case highlights the rarity and broad clinical spectrum of Hallopeau-type pemphigus vegetans. Its exuberant and polymorphic mucocutaneous manifestations may mimic infectious or inflammatory dermatoses and contribute to diagnostic difficulty. Recognition of this uncommon variant, supported by histopathology and immunofluorescence findings, can aid accurate diagnosis and timely management.

## Conclusions

This case suggests that a pustular onset evolving into extensive flexural vegetations with pan-ungual involvement may provide useful diagnostic clues to Hallopeau-type pemphigus vegetans. Recognition of this rare and distinctive presentation may facilitate timely diagnostic confirmation.
